# Distributed Data Classification with Coalition-Based Decision Trees and Decision Template Fusion

**DOI:** 10.3390/e27121205

**Published:** 2025-11-27

**Authors:** Katarzyna Kusztal, Małgorzata Przybyła-Kasperek

**Affiliations:** 1 Institute of Computer Science, University of Silesia in Katowice, Bȩdzińska 39, 41-200 Sosnowiec, Poland; katarzyna.kusztal@us.edu.pl; 2 Department of Informatics, Faculty of Natural Sciences and Informatics, Constantine the Philosopher University in Nitra, Tr. A. Hlinku 1, 949 01 Nitra, Slovakia

**Keywords:** distributed data, classification, decision trees, hierarchical system, conflict analysis, coalition formation, decision templates, interpretability

## Abstract

In distributed data environments, classification tasks are challenged by inconsistencies across independently maintained sources. These environments are inherently characterized by high informational uncertainty. Our framework addresses this challenge through a structured process designed for the reduction of entropy in the overall decision-making process. This paper proposes a novel framework that integrates conflict analysis, coalition formation, decision tree induction, and decision template fusion to address these challenges. The method begins by identifying compatible data sources using Pawlak’s conflict model, forming coalitions that aggregate complementary information. Each coalition trains a decision tree classifier, and the final decision is derived through decision templates that fuse probabilistic outputs from all models. The proposed approach is compared with a variant that does not use coalitions, where each local source is modeled independently. Additionally, the framework extends previous work based on decision rules by introducing decision trees, which offer greater modeling flexibility while preserving interpretability. Experimental results on benchmark datasets from the UCI repository demonstrate that the proposed method consistently outperforms both the non-coalition variant and the rule-based version, particularly under moderate data dispersion. The key contributions of this work include the integration of coalition-based modeling with decision trees, the use of decision templates for interpretable fusion, and the demonstration of improved classification performance across diverse scenarios.

## 1. Introduction

Contemporary systems for data processing and analysis operate in environments where information originates from multiple independently managed sources. Such fragmentation is a natural consequence of the organization of institutions, business processes, or research units collecting and storing their records according to internal procedures. On the one hand, this provides flexibility and allows data collection methods to be tailored to specific needs and conditions; on the other, it introduces serious challenges. Individual sources may represent reality differently–by applying distinct measurement protocols, storing datasets in heterogeneous formats, or maintaining varying levels of detail. As a result, simple aggregation is often impossible or leads to contradictory conclusions, which undermines the reliability of analyses. Consequently, the integration of distributed data has become one of the central concerns of modern data science.

This problem is universal and arises across many practical domains. In medicine, patient records are often stored in different healthcare providers, each relying on its own diagnostic procedures and laboratory tests. As a result, examination outcomes and the medical decisions derived from them may vary between hospitals. It is not uncommon for the same patient to be treated in more than one facility, which can lead to inconsistencies and discrepancies in diagnoses. In the financial sector, independent institutions maintain separate databases, where information about the same client may be differ depending on the applied risk assessment methods, directly influencing credit and investment decisions. Comparable difficulties also occur in business, where individual company branches analyze sales locally, focusing on the specifics of their market. This local perspective hinders the creation of a coherent organizational picture and limits the ability to make effective strategic decisions.

In response to these difficulties, the literature presents a variety of approaches to distributed data classification [1,2]. Broadly, they can be divided into interpretable models, such as decision trees and rule-based classifiers [3,4,5,6], and black-box models, including neural networks and deep learning techniques [7]. While the latter often achieve very high accuracy, the lack of explainability limits their usefulness in applications that require transparency of the classification process. Ensemble methods such as bagging, boosting, and stacking [8,9] represent another important research direction. However, these techniques mainly focus on improving predictive performance rather than integrating knowledge across multiple sources. More recently, federated learning has gained attention [10,11], allowing decentralized model training while preserving the privacy of local data. Although this approach addresses increasing demands for data protection, it typically relies on complex black-box models, without providing interpretability. Another line of research involves hierarchical classification schemes [12], where local models are combined within a higher-level structure; however, these methods do not directly resolve the issue of divergent predictions between sources. Managing uncertainty in multi-source and distributed environments has been a central topic in information fusion and decision-making research. Among the most influential frameworks, Dempster–Shafer evidence theory provides a flexible approach for representing and combining uncertain information, extending classical probability theory by allowing belief assignments to subsets of hypotheses rather than singletons [13,14]. This property makes Dempster–Shafer theory particularly suitable for applications in sensor fusion, fault diagnosis, and risk analysis [15,16,17]. However, the transformation of basic probability assignments into actionable probability distributions remains a critical challenge. Traditional methods such as the pignistic probability transformation redistribute mass uniformly across focal elements [18], while optimization-based approaches aim to minimize entropy for improved decisiveness [19]. Recent research introduces graph-based models to capture structural relationships among focal elements. For example, the ordered visibility graph probability method constructs a directed graph based on basic probability assignments ordering [20], while its weighted variant integrates belief entropy to improve interpretability [21]. Paper [17] proposes an enhanced probabilistic transformation using weighted visibility graph networks combined with advanced entropy measures, such as Jiroušek’s decomposable entropy [22]. Comparative studies show that these methods outperform classical probability transformation. Consequently, this research aligns with the fundamental principles of information theory, specifically focusing on maximizing information gain through classification model synergy and minimizing overall decision entropy across the distributed architecture.

Against this background, methods for the formal analysis of conflicts in distributed data are gaining growing relevance. One of the foundation approaches is Pawlak’s conflict analysis model [23], which enables the identification and description of dependencies between agents. This idea was subsequently extended, among others, within the framework of rough set theory [24], and also linked to three-way decision theory [25]. A study [26] demonstrated the possibility of broadening classical models by considering analysis across two universes. More recent research [27] has also shown that the approach can be applied in hierarchical systems, where scenarios of disagreement are constructed using methods inspired by cluster analysis.

In parallel, research has advanced on methods fusing classification results, aimed at producing a consistent decision from the predictions of multiple local models. The simplest strategies include majority voting and averaging, but these typically overlook relationships between classifiers and fail to capture more complex decision patterns. To overcome this limitations, decision templates were proposed by Kuncheva [28], which capture the characteristic behavior of ensembles and serve as a reference for evaluating new cases.

This paper introduces a new approach to the classification of distributed data, which combines conflict analysis, the construction of tree-based models, and the mechanism of decision templates. In the first stage, data sources are grouped into coalitions, enabling their collaboration and better utilization of complementary information. Next, decision trees are trained for each coalition. The final component is the application of decision templates, which provide stable result integration and robustness against local inconsistencies.

In the authors’ earlier study [29], conflict analysis was integrated with rule-based classification. Decision rules were induced using four algorithms: exhaustive search, covering, genetic, and LEM2. For classification purposes, three alternative strategies were applied: (1) the choice of the class of the first matching rule, (2) the assignment of the most frequent decision among matching rules, and (3) the selection of the class with the highest sum of covering rule weights. The analysis focused on comparing different induction-classification configurations. In a subsequent work [30], the same rule-based framework was further developed, where the classification process relied on the decision template mechanism. Building on this line of research, the current study extends the coalition- and template-based framework to decision trees. Replacing rules with trees broadens the ability to model complex, hierarchical dependencies between attributes, offering greater flexibility and integration potential, while preserving the interpretability of the classification process.

The contribution of this work is threefold:

- A novel framework for distributed data classification that integrates coalition formation based on conflict analysis with decision tree induction and decision template fusion.
- An interpretable modeling approach, where decision trees are used instead of rule-based classifiers, enabling the representation of complex attribute dependencies while maintaining transparency.
- A robust fusion mechanism, which leverages decision templates to integrate predictions from multiple coalition-based models, improving classification accuracy and consistency across diverse data sources.

The organization of the paper is as follows. Section 2 presents the proposed method, covering the construction of local models and the use of decision templates. The datasets and the experimental procedure are described as well. Section 3 reports and analyzes the obtained results. The implications of the findings and the study’s limitations are discussed in Section 4. Finally, Section 5 concludes with a summary of contributions and prospects for future work.

## 2. Materials and Methods

This section introduces the data representation used in the study and the proposed framework for distributed data. It also includes an illustrative example to demonstrate the operation of the method, followed by the description of the experimental setup used for evaluation.

### 2.1. Data Representation and Notation

Formally, we assume that the distributed data are represented as a set of local decision tables $T=\{T_{i}:i\in \left\{1,\ldots ,n\right\}\}$. Each $T_{i}=\left(U_{i},A,d\right)$ consists of a set of objects $U_{i}$, a set of conditional attributes *A*, and a decision attribute *d*. Within this study, the local tables are considered to be described by the same set of conditional attributes. As they originate from the same domain, the decision attribute is also common to all of them. This formalization ensures a consistent basis for further analysis and enables subsequent modeling and classification procedures.

### 2.2. Proposed Classification Framework

The proposed method can be outlined in four main stages:

1. Forming coalitions of sources using conflict analysis;
2. Combining data within each coalition and training a decision tree model on the aggregated set;
3. Deriving prediction vectors for training instances and generating a decision template corresponding to each decision class;
4. Conducting the final classification, where prediction vectors of test samples are matched against the decision templates using normalized Euclidean distance.

**Figure 1:**
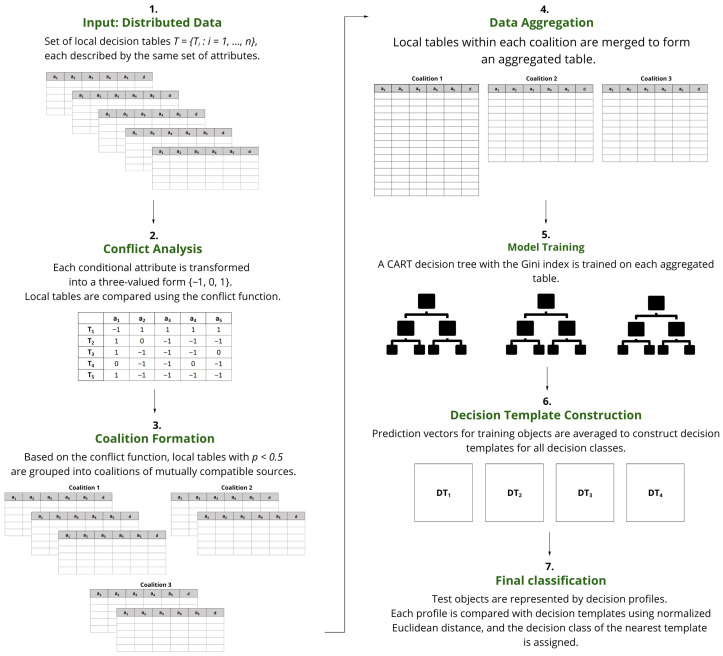
Workflow of the proposed classification framework for distributed data.

The workflow of the proposed framework is summarized in Figure 1.

Initially, coalitions of local tables are identified using Pawlak’s conflict analysis model [23]. For this purpose, each conditional attribute is expressed in a simplified form by assigning it one of three values from the set {−1,0,1}. This transformation provides a uniform representation of local tables, which is then used for constructing the information system S=(T,A). The assignment procedure differs depending on the type of attribute.

The use of simple quantisation into {−1,0,1} follows the original formulation of Pawlak’s conflict analysis model [23], which emphasizes symbolic representation and interpretability over numerical precision. This approach enables a clear and intuitive comparison of local data sources by reducing attribute values to a common scale of deviation from the global norm. While more nuanced encoding or adaptive discretisation methods could retain richer data characteristics, they often introduce additional complexity and may obscure the interpretability of the conflict relations. In contrast, the three-valued representation preserves the transparency of the model and aligns with the foundational principles of conflict analysis in distributed environments.

For each quantitative attribute $a_{quan}\in A$, we assign to every local table $T_{i}$ its mean value, written as ${\bar{Val}}_{a_{quan}}^{i}$. Subsequently, the global mean ${\bar{Val}}_{a_{quan}}$ and the global standard deviation $SD_{a_{quan}}$ are calculated over the entire collection of tables. Based on these statistics, we introduce a mapping $a_{quan}:T\to \left\{-1,0,1\right\}$, specified as follows:

(1) $$a_{quan}\left(T_{i}\right)=\left\{\begin{array}{cc} 1 & \mathrm{if}\;\;\;{\bar{Val}}_{a_{quan}}+SD_{a_{quan}}<{\bar{Val}}_{a_{quan}}^{i}\\ 0 & \mathrm{if}\;\;\;{\bar{Val}}_{a_{quan}}-SD_{a_{quan}}\leq {\bar{Val}}_{a_{quan}}^{i}\leq {\bar{Val}}_{a_{quan}}+SD_{a_{quan}}\\ -1 & \mathrm{if}\;\;\;{\bar{Val}}_{a_{quan}}^{i}<{\bar{Val}}_{a_{quan}}-SD_{a_{quan}} \end{array}\right.$$

A value of 0 indicates that the attribute in the considered table remains within the typical range observed across all tables. A value of 1 means that it exceeds the global tendency, whereas −1 corresponds to lower-than-usual values.

In contrast, for a qualitative attribute $a_{qual}\in A$, we describe its distribution within each local table $T_{i}$. If $a_{qual}$ admits *c* distinct categories $val_{1},\ldots ,val_{c}$, we define the vector $Val_{a_{qual}}^{i}=\left(n_{1}^{i},\ldots ,n_{c}^{i}\right)$, where each component $n_{j}^{i}$ denotes the number of objects in $T_{i}$ taking the value $val_{j}$. Each vector is then normalized. Subsequently, to reduce this representation, the 3-means clustering algorithm with Euclidean distance is applied to the set of such vectors. The obtained centroids are then sorted in descending order according to the value of their first coordinate. The clusters are assigned the values 1, 0, and −1, respectively. As a result, three groups of tables are obtained, characterized by similar distributions of attribute values.

With conditional attributes represented in the three-valued form, we define a conflict function ρ:T×T→[0,1] describing the relation of two local tables. It is given by

(2) $$\rho \left(T_{i},T_{j}\right)=\frac{card\{a\in A:a\left(T_{i}\right)\neq a\left(T_{j}\right)\}}{card\{A\}}.$$

The value of $\rho (T_{i},T_{j})$ corresponds to the proportion of attributes on which the tables disagree. A lower value indicates higher similarity, while a higher value reflects stronger divergence. Tables satisfying $\rho (T_{i},T_{j})<0.5$ are considered compatible and are grouped into the coalition. Hence, each coalition consists of tables that are mutually consistent in at least half of the attributes.

The compatibility threshold of 0.5 originates from Pawlak’s original conflict analysis model [23], which interprets agreement in more than half of the attributes as sufficient for establishing compatibility. This simple and intuitive criterion ensures interpretability and aligns with the foundational principles of conflict-based reasoning. Previous studies introduced parameterized extensions with adjustable thresholds to give more flexibility in coalition formation. For example, ref. [31] examined the effect of such parameters in a dispersed data classification framework based on allied relations, which is conceptually different from the approach used in this work. While the present study adopts the classical threshold for consistency with the original model, future work will explore parameterized variants to assess their influence on coalition structure and classification performance.

Next, an aggregation decision table $T_{j}^{aggr}=\left(U_{j}^{aggr},A,d\right)$ is created by merging the data from all local tables belonging to that coalition. The universe $U_{j}^{aggr}$ is defined as the union of objects from the constituent tables. The sets of conditional attributes *A* and the decision attribute *d* are retained from the original representation. For every object $x\in U_{i}$, the corresponding attribute values in the aggregated table are obtained directly from its source table $T_{i}$.

For each coalition, a decision tree model following the classification and regression tree (CART) algorithm with the Gini index as the splitting criterion [32] is trained on the aggregated table $T_{j}^{aggr}$. Although the Gini index is used, it is a measure of impurity functionally related to the concept of information entropy often employed in decision tree induction. Both measures aim to achieve the maximum information gain (i.e., the largest possible reduction in classification uncertainty) at each node split, thereby explicitly grounding the model construction in information theory principles. Entropy and information gain will be utilized in future research as key criteria for determining the optimal partitioning of tree structures. The Gini index was selected due to its computational efficiency and its proven effectiveness in classification tasks involving dispersed data. In particular, previous research has shown that the Gini index performs comparably to other criteria such as entropy and twoing in distributed environments. For example, ref. [33] conducted a comparative study of splitting criteria for decision trees applied to dispersed data, demonstrating that while entropy and twoing offer alternative perspectives on impurity, the Gini index remains a robust and interpretable choice. Its simplicity and speed make it especially suitable for large-scale distributed systems, which aligns with the goals of this framework.

Since the decision template fusion method [28] operates on probability-based predictions, each classifier produces outputs at the measurement level for both training and test objects. For an object *x*, these probabilities are obtained in Python 3.13.0 using the predict_proba function from the scikit-learn library [34] and are represented as a normalized vector $[\mu_{j,1}\left(x\right),\ldots ,\mu_{j,i}\left(x\right),\ldots ,\mu_{j,c}\left(x\right)],$ where *c* denotes the number of decision classes.

In the next step, probability outputs from all coalition-based classifiers are used to construct decision templates. The process of constructing templates $DT_{i}$ by averaging prediction vectors (Equation (3)) serves as a fusion mechanism designed to minimize the collective uncertainty (or output entropy) of the ensemble. By integrating probabilistic outputs from all coalition models, this approach effectively extracts a consensus informational profile, which increases the stability and reduces the entropy of the final classification decision compared to individual local predictions. For each decision class *i*, a template $DT_{i}$ is built by averaging the prediction vectors of training objects that belong to this class:  

(3) $$DT_{i}=\frac{1}{card\{X_{i}\}}\sum_{x\in X_{i}}\left[\begin{array}{ccccc} \mu_{1,1}\left(x\right) & \ldots & \mu_{1,i}\left(x\right) & \ldots & \mu_{1,c}\left(x\right)\\ \; & \; & \ldots \\ \mu_{j,1}\left(x\right) & \ldots & \mu_{j,i}\left(x\right) & \ldots & \mu_{j,c}\left(x\right)\\ \; & \; & \ldots \\ \mu_{L,1}\left(x\right) & \ldots & \mu_{L,i}\left(x\right) & \ldots & \mu_{L,c}\left(x\right) \end{array}\right],$$

where $X_{i}$ denotes the set of training objects labeled with class *i*, and *L* is the number of coalition-based models.

To classify a new (test) object $\bar{x}$, a decision profile is generated from its probability predictions across all classifiers:

(4) $$DP\left(\bar{x}\right)=\left[\begin{array}{ccccc} \mu_{1,1}\left(\bar{x}\right) & \ldots & \mu_{1,i}\left(\bar{x}\right) & \ldots & \mu_{1,c}\left(\bar{x}\right)\\ \; & \; & \ldots \\ \mu_{j,1}\left(\bar{x}\right) & \ldots & \mu_{j,i}\left(\bar{x}\right) & \ldots & \mu_{j,c}\left(\bar{x}\right)\\ \; & \; & \ldots \\ \mu_{L,1}\left(\bar{x}\right) & \ldots & \mu_{L,i}\left(\bar{x}\right) & \ldots & \mu_{L,c}\left(\bar{x}\right) \end{array}\right].$$

The final decision is made by comparing the decision profile $DP(\bar{x})$ with each decision template $DT_{i}$ using the normalized Euclidean distance:

(5) $$s\left(DP\left(\bar{x}\right),DT_{i}\right)=\frac{1}{L\cdot c}\sum_{m=1}^{L}\sum_{l=1}^{c}{\left(DP^{m,l}\left(\bar{x}\right)-DT_{i}^{m,l}\right)}^{2},$$

where $DP^{m,l}\left(\bar{x}\right)$ and $DT_{i}^{m,l}$ refer to the values at the *m*-th row and *l*-th column of $DP(\bar{x})$ and $DT_{i}$, respectively. The object $\bar{x}$ is then assigned to the class whose template yields the smallest distance, indicating the closest match between the prediction patterns.

For clarity, the pseudo-code of the proposed framework is presented in Algorithm 1. The computational complexity can be determined by analyzing the operations carried out within its individual components. The creation of the information system involves deriving summary statistics for all conditional attributes across the collection of local tables. This operation requires O((N+n)·m) time, where $N=\sum_{i=1}^{n}card\left\{U_{i}\right\}$, n=card{T}, and m=card{A}. Because typically N≥n, the contribution of the *n*-dependent part is marginal relative to the effort associated with processing all objects. Pairwise conflict function values are next obtained by comparing the three-valued attribute representations of each pair of local tables. With n(n−1) ordered pairs, this stage scales as $O(n^{2}\cdot m)$. Coalition formation proceeds by identifying subsets of local tables that satisfy the compatibility condition (ρ<0.5); in the worst case, determining all admissible groupings entails examining all subsets of the *n* tables, leading to an exponential upper bound of $O(2^{n})$. After the coalition structure has been established, the data within each coalition are aggregated by concatenating the objects originating from its constituent local tables. Across the framework, this operation is linear in the total number of instances, yielding O(N). Training a CART model for each coalition requires $O(N_{j}\cdot m\log N_{j})$ for a dataset of size $N_{j}=card\left\{U_{j}^{aggr}\right\}$. Summing these contributions over all coalitions gives a total cost of $O\left(\sum_{j}N_{j}\cdot m\log N_{j}\right)$. Since $N_{j}\leq N$ for all *j*, this term can be upper-bounded by $O\left((\sum_{j}N_{j})\cdot m\log N\right)$. Decision templates are then constructed by averaging the prediction vectors produced by the *k* coalition models over the training objects assigned to each decision class. Since each probability vector contains *c* components, this stage runs in O(N·k·c). Finally, classifying a new object consists in generating its decision profile from the predictions of all coalition-based models and comparing it with each decision template. Each distance computation takes O(k·c), so the classification of a single object proceeds in $O(k\cdot c^{2})$.

Taken together, the overall computational complexity of the proposed framework is dominated by the exponential cost of coalition formation, while all remaining stages operate in polynomial or linear time. In practical scenarios, however, compatible coalitions tend to emerge earlier in the process, so only part of the possible subsets is explored. Thus, the final coalition structure is obtained faster than the theoretical upper bound suggests.
**Algorithm 1** Pseudo-code of the proposed classification framework for distributed data**Input:** A set of local decision tables $T={\left\{T_{i}=\left(U_{i},A,d\right)\right\}}_{i=1}^{n}$.**Output:** Final classification result for a test object $\bar{x}$.*Creation of information system*for each conditional attribute a∈A, define the function $a\left(T_{i}\right)\in \left\{-1,0,1\right\}$:   if *a* is quantitative then     Use Equation (1)   else     Apply the procedure described for qualitative attributesForm the information system S=(T,A).*Coalition formation*for each pair $(T_{i},T_{j})\in T\times T$:   Use Equation (2) to compute the conflict function value $\rho (T_{i},T_{j})$Group local tables into coalitions $C_{1},\ldots ,C_{k}$ (where *k* denotes the number of coalitions) so that tables within each coalition satisfy $\rho (T_{i},T_{j})<0.5$.*Data aggregation*for each coalition $C_{j}$:   Combine local tables into the aggregated table $T_{j}^{aggr}=\left(U_{j}^{aggr},A,d\right)$*Model training*for each aggregated table $T_{j}^{aggr}$:   Train a CART decision tree model $CT_{j}^{aggr}$ using the Gini index*Construction of decision templates*for each training object *x*:   for each coalition model $CT_{j}^{aggr}$:      Obtain the class-probability vector $\mu_{j}\left(x\right)=\left[\mu_{j,1}\left(x\right),\ldots ,\mu_{j,i}\left(x\right),\ldots ,\mu_{j,c}\left(x\right)\right]$ (where *c* is the number of decision classes)for each decision class *i*:   Average prediction vectors of training objects belonging to class *i* to generate the decision template $DT_{i}$ according to Equation (3)*Final classification*for a (new) test object $\bar{x}$:   for each coalition model $CT_{j}^{aggr}$:      Obtain the class-probability vector $\mu_{j}\left(\bar{x}\right)=\left[\mu_{j,1}\left(\bar{x}\right),\ldots ,\mu_{j,i}\left(\bar{x}\right),\ldots ,\mu_{j,c}\left(\bar{x}\right)\right]$   Form the decision profile $DP(\bar{x})$ using Equation (4)   Compute normalized Euclidean distances $s(DP\left(\bar{x}\right),DT_{i})$ as defined in Equation (5)   Return the decision class *i* corresponding to the smallest distance $s(DP\left(\bar{x}\right),DT_{i})$

### 2.3. Illustrative Example

To demonstrate the operation of the proposed framework, consider a symbolic, practice-oriented example reflecting a business environment. Three local decision tables, denoted as $T_{1}$, $T_{2}$, and $T_{3}$, represent customer purchasing activity recorded by different regional branches, as summarized in Table 1. All local tables share the same set of conditional attributes $A=\{a_{1},a_{2},a_{3}\}$ and a common decision attribute *d*. Attributes $a_{1}$ and $a_{2}$ are quantitative, while $a_{3}$ is qualitative. Specifically, $a_{1}$ denotes the customer’s average number of transactions per month, $a_{2}$ represents the average purchase value (expressed in relative units), and $a_{3}$ indicates the dominant shopping channel (online, retail, or business). The decision attribute *d* indicates the customer satisfaction level, where 1 corresponds to low and 2 to high satisfaction. This dataset is entirely synthetic and was designed solely for explanatory purposes.

Following the procedure described in Section 2.2, all conditional attributes are transformed into the three-valued representation {−1,0,1} according to Pawlak’s conflict analysis model. This process varies depending on whether the attribute is quantitative or qualitative. Table 2 presents the resulting information system obtained for the local tables $T_{1}$, $T_{2}$, and $T_{3}$.

Based on the information system, the conflict function is computed in line with Pawlak’s model. The generated conflict matrix is shown in Table 3, where each element quantifies the degree of disagreement between two local tables in terms of their symbolic attribute representations.

With the threshold ρ<0.5, local tables $T_{1}$ and $T_{2}$ are compatible and form coalition $C_{1}=\left\{T_{1},T_{2}\right\}$, whereas $T_{3}$ remains separate as $C_{2}=\left\{T_{3}\right\}$. For each coalition, data are aggregated and a CART decision tree is constructed using the Gini index.

Afterward, the coalition-based classifiers produce class-probability predictions for two decision classes (c=2) and two coalitions (L=2). In accordance with Equation (3), averaging the predictions across training objects within each decision class yields two decision templates, $DT_{1}$ and $DT_{2}$, associated with classes 1 and 2, respectively:

$$DT_{1}=\left[\begin{array}{cc} 0.80 & 0.20\\ 0.40 & 0.60 \end{array}\right],\;DT_{2}=\left[\begin{array}{cc} 0.25 & 0.75\\ 0.00 & 1.00 \end{array}\right].$$

As can be seen, in $DT_{1}$ the first coalition assigns a higher probability to class 1 (0.80 vs. 0.20), indicating a stronger association with this class. In contrast, the second coalition within $DT_{1}$ shows a higher probability for class 2 (0.60 vs. 0.40), suggesting partial disagreement between coalitions in terms of class preference. In $DT_{2}$, both coalitions assign higher probabilities to class 2, which indicates a more consistent representation of this class across the coalitions.

For a test object $\bar{x}$ described by the attributes $a_{1}=9$, $a_{2}=11$, and $a_{3}=\mathrm{retail}$, the prediction profile obtained across the coalitions is given by:

$$DP\left(\bar{x}\right)=\left[\begin{array}{cc} 1.00 & 0.00\\ 0.00 & 1.00 \end{array}\right].$$

The first coalition assigns the test object $\bar{x}$ entirely to class 1, while the second coalition assigns it to class 2, providing conflicting predictions. To determine the final classification, the similarity between the decision profile and each decision template is computed using the normalized Euclidean distance (Equation (5)). The resulting distances are:

$$s\left(DP\left(\bar{x}\right),DT_{1}\right)=0.1,\;s\left(DP\left(\bar{x}\right),DT_{2}\right)=0.28125.$$

As the smaller distance reflects greater similarity between the decision profile and the corresponding decision template, the test object $\bar{x}$ is assigned to class 1. Interpreted in business terms, the model predicts that the customer belongs to the low-satisfaction group.

### 2.4. Experimental Setup and Evaluation Procedure

The experiments, intended to assess the effectiveness of the proposed method, were performed on three datasets—Balance Scale, Vehicle Silhouettes [35], and Car Evaluation [36]—all sourced from the UCI Machine Learning Repository [37]. Each dataset was divided into two non-overlapping subsets using a stratified sampling strategy: 70% of the instances were assigned to the training set, while the remaining 30% formed the test set. The Balance Scale dataset includes 625 instances, with 437 used for training and 188 for testing, defined by four categorical attributes and three decision classes representing balance states: B (balanced), L (titled left), and R (titled right). The Vehicle Silhouettes dataset contains 846 instances, of which 592 were used for training and 254 for testing. These are described by 18 numerical attributes and grouped into four decision classes corresponding to different vehicle types: bus, opel, saab, and van. Finally, the Car Evaluation dataset comprises 1728 records (1209 for training and 519 for testing), characterized by six categorical attributes and four decision labels reflecting car acceptability levels: unacc (unacceptable), acc (acceptable), good, and vgood (very good).

Although the data are not originally distributed, they were divided into several local tables to simulate the presence of multiple data sources that describe the same decision problem. Four configurations were tested, with 5, 7, 9, and 11 local tables. These settings provide a gradual increase in data fragmentation, allowing the analysis of the method’s robustness and coalition formation behavior. Consequently, twelve versions of the datasets were generated. All local tables retained the complete set of attributes but contained only a portion of the training data. The stratified sampling ensured that, for each decision class, instances were proportionally assigned to the tables. Such a setup enables meaningful comparison and coalition formation, as the observed differences between sources result from the intrinsic properties of the data rather than from variations in class composition.

Classification performance was evaluated on the test set of each dataset. A diverse set of indicators was used to provide a multidimensional view of the results, namely accuracy (Acc), balanced accuracy (BAcc), precision (Prec.), recall, F-measure (F.m.), and geometric mean (G-mean). In general, accuracy expresses how many observations were correctly assigned to their true categories. When focusing on specific classes, precision measures the correctness of positive predictions, while recall indicates the proportion of real class members that were successfully detected. Their joint behavior is captured by the F-measure, calculated as the harmonic mean of these two quantities:

(6) $$F-\mathrm{measure}=2\cdot \frac{\mathrm{Precision}\cdot \mathrm{Recall}}{\mathrm{Precision}+\mathrm{Recall}}.$$

Since the analyzed datasets differ in class distribution, additional measures were incorporated. Balanced accuracy summarizes the average recall obtained for all classes, while G-mean evaluates the uniformity of performance, rewarding models that achieve comparable recall across categories.

The experiments were structured according to the main principles of the proposed framework, and consisted of the following steps:

- Creation of coalitions among local decision tables;
- Training decision tree models using the data combined within each coalition;
- Construction of prediction vectors for training and test instances with reference to the built decision trees;
- Generation of class-specific decision templates;
- Final classification of test samples based on normalized Euclidean distance to the decision templates.

To provide a reference for performance evaluation, a baseline approach was also implemented. In this variant, conflict analysis and coalition formation were omitted, and each local table was used to train an independent decision tree model.

## 3. Results

This section presents the obtained results and their analysis. The evaluation is structured around three main aspects. The first part concerns the performance and execution time of the proposed method in comparison with the baseline approach, the second addresses its interpretability through the examination of the generated decision templates, and the third focuses on the findings in relation to those achieved for the rule-based variant of this framework [30].

### 3.1. Comparison with the Baseline Approach

As presented in Table 4, the proposed approach generally yields higher classification results than the baseline method. In the case of the Balance Scale dataset, results for 5 local tables are not included due to the absence of coalitions in this configuration. For other tested dispersion levels, the proposed framework consistently outperforms the baseline across all dispersion levels, achieving higher values in most evaluation metrics. This observation highlights the robustness of the method, which demonstrates stable and reliable predictive performance for this dataset.

In contrast, the findings for the remaining data collections reveal a more diverse pattern. While the proposed approach often surpasses the baseline, its performance varies with the level of data dispersion. The framework tends to produce stronger results under moderate dispersion, suggesting that coalition formation is particularly effective when each local table retains sufficient information for meaningful conflict analysis. Moreover, metrics such as F-measure and G-mean follow a similar trend, confirming that the proposed method provides more balanced predictions across decision classes. As the degree of dispersion increases and local tables become smaller and less representative, a slight decline in performance can be observed.

**Figure 2:**
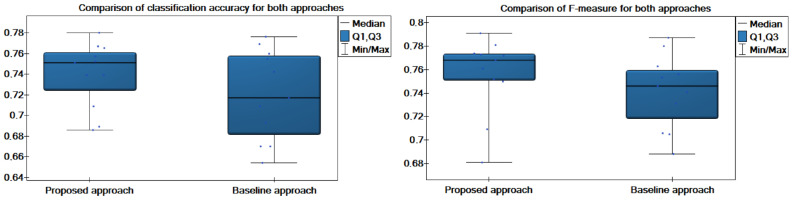
Comparison of accuracy and F-measure obtained for the proposed and the baseline approaches.

Figure 2 presents box plots comparing the distribution of classification accuracy and F-measure for the proposed coalition-based approach and the approach without coalition formation. The plots reveal that the coalition-based method achieves higher median values for both metrics (0.751 for accuracy and 0.768 for F-measure) compared to the non-coalition variant (0.717 and 0.746, respectively). Moreover, the interquartile range for the proposed approach is narrower, indicating greater stability and less variability across different data dispersion scenarios. The lower whisker for the non-coalition approach extends to substantially smaller values, suggesting that this method is more sensitive to unfavorable configurations of distributed data.

Additionally, to complement the theoretical complexity analysis, the execution time of the proposed framework was compared with that of the baseline model. All computations were performed on a portable computer equipped with an AMD Ryzen 5 4600H processor, 32 GB RAM and Microsoft Windows 11. The algorithms were implemented in Python. Table 5 reports the exact running times (in seconds) for all three datasets and dispersion levels. Across all configurations, the proposed method consistently runs faster than the baseline, with differences becoming more pronounced as the number of local tables increases. These results demonstrate that, despite the exponential worst-case complexity of coalition formation, the observed runtime stays within practical limits for all tested settings (up to 11 local tables).

### 3.2. Interpretability Analysis

An important feature of the proposed approach is its interpretability, which allows for a detailed examination of how individual local models contribute to the final class assignment. This property makes it possible to identify patterns of specialization and the impact of coalition-based aggregation. To illustrate this aspect and provide a clearer insight into the behavior of the ensemble framework, Table 6, Table 7 and Table 8 present exemplary decision templates corresponding to one selected dispersion level for each dataset. The configurations were chosen based on the largest observed difference in classification accuracy between the proposed and baseline approaches, making them the most informative for further analysis. Specifically, the templates were obtained for the following numbers of local tables: 7 for Balance Scale, 5 for Vehicle Silhouettes, and 7 for Car Evaluation. In the tables, decision templates are denoted by the class labels (e.g., $DT_{B}$ for class B), which are denoted as $DT_{i}$ in Equation (3). Each local model $CT_{j}^{aggr}$ is derived, in the proposed approach, from the aggregated table $T_{j}^{aggr}$ formed within a coalition, whereas in the baseline, $CT_{i}$ originates from the individual table $T_{i}$. The values in the columns *p*(class) represent the averaged class membership probabilities $\mu_{j,i}\left(x\right)$ for each local model. For the chosen dispersion levels, the following coalitions were formed in the proposed method:

- Balance Scale (7 local tables, 4 coalitions): {$T_{2},T_{4},T_{5},T_{6}$}, {$T_{1}$}, {$T_{7}$}, {$T_{3}$};
- Vehicle Silhouettes (5 local tables, 4 coalitions): {$T_{1},T_{5}$}, {$T_{4}$}, {$T_{3}$}, {$T_{2}$};
- Car Evaluation (7 local tables, 6 coalitions): {$T_{4},T_{5}$}, {$T_{2},T_{5}$}, {$T_{2},T_{6}$}, {$T_{1}$}, {$T_{7}$}, {$T_{3}$}.

As can be observed for the Balance Scale dataset, the most noticeable difference between the two approaches appears in the decision template corresponding to class B. In the baseline approach, none of the local models assign the highest probability to this class; instead, the dominant probabilities are associated with other classes. In contrast, in the proposed approach, $CT_{1}^{aggr}$ clearly indicates class B with the highest probability (0.588), demonstrating the emergence of a localized specialization for this class. This effect is directly linked to the coalition underlying $CT_{1}^{aggr}$, which aggregates information from multiple local tables and strengthens the model’s ability to capture class-specific patterns. For the templates $DT_{L}$ and $DT_{R}$, both approaches show generally consistent results. However, the proposed approach produces sharper probability peaks (e.g., 0.955 for $CT_{1}^{aggr}$ in $DT_{R}$ compared to 0.871 for $CT_{5}$ in the baseline), reflecting stronger class assignment within coalitions. For the Vehicle Silhouettes dataset, the proposed approach also reinforces class assignment, but the differences between the two approaches are more subtle. This is due to the fact that only one coalition is multi-table, while the remaining coalitions consist of single tables. Notably, for the Car Evaluation dataset, more pronounced enhancement effects are observed, particularly for the templates corresponding to classes vgood and good, where the average probability increases from 0.482 to 0.585 and from 0.474 to 0.505, respectively.

These observations confirm that the decision templates offer a transparent perspective, highlighting the role of coalition structures in shaping the final classification outcomes. In the baseline approach, each model is trained on an individual local table, which may lead to fragmented class patterns. In contrast, coalition-based models, built on merged and compatible data sources, capture more coherent decision tendencies, resulting in clearer and more stable class-related behaviors, thus improving interpretability.

### 3.3. Comparison with Rule-Based Models

To better understand the effect of the chosen local modeling strategy, the proposed tree-based approach was also compared with its variant relying on decision rule induction [30]. Table 9 summarizes the classification results obtained with rule-based models for each dataset and dispersion level. These results come directly from [30], which used the same datasets as in the present study. In that work, four rule induction algorithms were considered: the exhaustive search algorithm, the covering algorithm, the genetic algorithm, and LEM2. For each configuration, the method achieving the highest classification accuracy was selected and reported in the table. The abbreviations Exh and Gen refer to the exhaustive search and genetic algorithms, respectively, while Exh/Gen indicates identical results for both methods. The last column, ΔAcc, shows the difference in accuracy (in percentage points) between the proposed tree-based framework and its rule-based counterparts. Positive values indicate that the tree-based approach outperformed the rule-based variant, whereas negative values correspond to the opposite case. For the Balance Scale dataset, results for 5 local tables are again not included due to the absence of coalitions in this configuration.

A closer look at the results reveals that the use of decision trees as local models often leads to better classification performance compared to rule-based models. The most evident differences are observed for the Vehicle Silhouettes and Car Evaluation datasets under moderate dispersion levels. In these configurations, higher values of ΔAcc are obtained–for instance, for Vehicle with 9 local tables, the accuracy increases by 0.079, corresponding to an 11.27% relative gain. In addition, the improvements are also visible in other evaluation metrics, including G-mean, balanced accuracy, and F-measure. For Vehicle, these effects appear across most dispersion levels (e.g., an increase in G-mean from 0.797 to 0.853 for 9 local tables), and for Car, particularly at lower dispersion levels, with G-mean improving in all tested configurations. These tendencies suggest that decision trees adapt better to the underlying structure of the data, resulting in more balanced predictions across decision classes. This, in turn, strengthens the coalition-based framework by improving its overall classification reliability, especially under favorable dispersion conditions.

## 4. Discussion

The experimental results demonstrate that the proposed tree-based framework often achieves better classification performance compared to both the baseline approach and the rule-based variant. The improvements are most visible when the local tables preserve enough informative structure to enable meaningful collaboration between models. This cooperation allows the models to better exploit complementary knowledge available across local sources. These outcomes highlight the potential of coalition-based mechanisms to enhance the robustness of the classification process and support more balanced and reliable decision-making in distributed data settings.

A key factor behind this improvement is the way coalition formation increases the representativeness of the training data and, consequently, the reliability of the classification process. By aggregating similar local tables into larger coalitions, the method reduces the negative impact of limited sample size and local variability. As a result, the decision tree models can better capture underlying patterns and improve their generalization capability. The use of decision templates also provides a clear and interpretable view of how coalition-level models contribute to the final classification outcome. This is particularly valuable in scenarios where transparency and explainability are essential.

Another important observation concerns the method’s behavior under different levels of data dispersion. The best results are achieved mainly at moderate dispersion, where the balance between local variability and shared structure is optimal for coalition formation. At this level, local tables contain enough distinct yet complementary information to make aggregation meaningful, while not being too fragmented to undermine the learning process. As dispersion increases, the representativeness of individual tables decreases, which naturally limits the gains from aggregation. In such settings, the performance of the method declines, but in a predictable manner, reflecting the reduced informational value of the local sources. Although other approaches may outperform it at the highest dispersion level, the proposed strategy maintains a reasonable level of effectiveness, highlighting its robustness to data fragmentation. These observations are consistent with information theory principles: lower uncertainty enables more efficient knowledge integration, while higher entropy constraints the capacity for meaningful collaboration between distributed models.

Although this study focuses exclusively on the Gini index, future work will explore the use of alternative splitting criteria such as entropy and twoing. These criteria may offer different insights into data structure and classification performance, particularly in the context of dispersed data. Comparative experiments involving multiple impurity measures could help identify optimal strategies for specific data distributions and application domains. Such extensions would enhance the generalizability and adaptability of the proposed approach.

An additional factor contributing to the effectiveness of the approach is the use of decision trees as local models. Their ability to capture complex relationships and produce well-structured decision boundaries enhances the quality of the coalition aggregation, leading to more coherent and informative decision templates. This suggests that the selection of flexible and expressive local models can play a crucial role in maximizing the benefits of coalition-based mechanisms in distributed classification settings.

Despite these promising results, the proposed approach has several limitations that should be acknowledged. First, its effectiveness depends on the number and size of the local tables. When the tables become too small or unbalanced, the benefits of coalition formation are reduced. Second, the method introduces an additional computational overhead associated with building and maintaining coalition structures, which may become relevant for large-scale applications. Although the theoretical complexity grows exponentially with the number of local tables, the empirical results (Section 3.1) indicate that the execution time remains reasonable even for the largest tested configuration (11 local tables). This suggests that the proposed approach is computationally feasible in moderately distributed environments and that its practical scalability is more favorable than implied by the theoretical analysis. Third, the current evaluation was conducted on a limited number of datasets and controlled data partitioning scenarios. Although these benchmark datasets allow for a transparent and interpretable assessment of the framework’s behavior, more complex, noisy, or domain-specific data will be considered in future work, as they are likely to require further methodological refinement to ensure scalability and robustness.

From a practical perspective, the proposed coalition-based framework may be particularly beneficial in scenarios where data are inherently fragmented or decentralized. Such settings frequently occur in areas where direct data integration is difficult or impossible due to technical, organizational, or legal constraints. By enabling more effective aggregation of knowledge from multiple sources without requiring full data centralization, the approach provides a flexible solution for improving classification performance in distributed environments. Furthermore, the use of decision templates supports a higher level of interpretability, which can be advantageous in domains where transparency and explainability are critical, such as healthcare, finance, or business.

## 5. Conclusions

This work introduced a distributed data classification method that integrates conflict analysis, coalition formation, decision tree induction, and decision template fusion. The proposed framework enables knowledge to be combined from multiple sources while keeping the resulting model transparent and effective across data sources. Furthermore, by utilizing the Gini index to maximize information gain during tree induction and employing Decision Templates to minimize decision entropy during final fusion, this framework offers a methodology that is deeply rooted in information theory for uncertainty management in decentralized systems.

The approach was experimentally evaluated on three benchmark datasets from the UCI Machine Learning Repository: Balance Scale, Vehicle Silhouettes, and Car Evaluation. Its performance was compared with a baseline method that does not involve coalition formation as well as with a rule-based variant proposed in previous work by the authors. The results demonstrated that the proposed method often achieved better classification performance, with the improvement observed at moderate levels of data dispersion. In addition, the study also analyzed the generated decision templates, showing that they strengthen the specialization of class predictions and provide a transparent representation of coalition-level behavior.

The proposed coalition-based approach provides a practical and transparent way to integrate distributed knowledge through partial aggregation of local data, without requiring full centralization. This makes it particularly relevant in domains such as healthcare, finance, or business, where information is often fragmented across multiple independent entities. By supporting more coherent and explainable decision-making, the method has the potential to address key challenges associated with decentralized data environments.

Future research will focus on extending the proposed approach to scenarios in which local tables are defined over partially different feature sets. Such an extension will make it possible to evaluate the robustness and adaptability of coalition-based mechanisms under more realistic and heterogeneous data conditions.

## Figures and Tables

**Table 1:** Example of local tables.

$T_{1}$				
$U_{1}$	$a_{1}$	$a_{2}$	$a_{3}$	*d*
$x_{1}$	8	10	online	1
$x_{2}$	9	10	online	1
$x_{3}$	8	11	retail	2
$T_{2}$				
$U_{2}$	$a_{1}$	$a_{2}$	$a_{3}$	*d*
$x_{1}$	9	11	retail	1
$x_{2}$	8	12	business	2
$x_{3}$	9	10	retail	2
$T_{3}$				
$U_{3}$	$a_{1}$	$a_{2}$	$a_{3}$	*d*
$x_{1}$	13	7	business	1
$x_{2}$	13	7	retail	1
$x_{3}$	14	8	business	2

**Table 2:** Information system.

	$a_{1}$	$a_{2}$	$a_{3}$
$T_{1}$	0	0	−1
$T_{2}$	0	0	0
$T_{3}$	1	−1	1

**Table 3:** Conflict matrix.

	$T_{1}$	$T_{2}$	$T_{3}$
$T_{1}$	0.00	0.33	1.00
$T_{2}$	0.33	0.00	1.00
$T_{3}$	1.00	1.00	0.00

**Table 4:** Results of classification accuracy (Acc), balanced accuracy (BAcc), precision (Prec.), recall, F-measure (F-m.), and geometric mean (G-mean) for the proposed and baseline approaches across datasets and dispersion levels.

Dataset	# Local	Proposed Approach	Baseline Approach
	Tables	Acc/BAcc/Prec./Recall/F-m./G-Mean	Acc/BAcc/Prec./Recall/F-m./G-Mean
Balance Scale	7	**0.739**/0.627/0.819/0.739/0.772/0.816	0.670/0.633/0.866/0.670/0.741/0.791
	9	**0.686**/0.644/0.867/0.686/0.752/0.801	0.654/0.676/0.893/0.654/0.731/0.788
	11	**0.739**/0.738/0.889/0.739/0.791/0.838	0.670/0.688/0.902/0.670/0.746/0.800
Vehicle Silhouettes	5	**0.752**/0.745/0.751/0.752/0.750/0.832	0.693/0.675/0.688/0.693/0.688/0.791
	7	0.709/0.691/0.714/0.709/0.709/0.804	0.709/0.696/0.710/0.709/0.705/0.804
	9	**0.780**/0.766/0.786/0.780/0.781/0.853	0.760/0.750/0.770/0.760/0.756/0.840
	11	0.689/0.673/0.682/0.689/0.681/0.787	**0.717**/0.698/0.707/0.717/0.706/0.807
Car Evaluation	5	**0.767**/0.670/0.786/0.767/0.774/0.779	0.755/0.697/0.777/0.755/0.763/0.765
	7	**0.765**/0.750/0.792/0.765/0.773/0.782	0.742/0.741/0.782/0.742/0.753/0.775
	9	0.751/0.702/0.783/0.751/0.761/0.775	**0.769**/0.769/0.810/0.769/0.780/0.811
	11	0.757/0.760/0.795/0.757/0.768/0.791	**0.776**/0.797/0.823/0.776/0.787/0.822

**Table 5:** Execution time (in seconds) of the proposed and baseline approaches across datasets and dispersion levels.

Dataset	# Local Tables	Proposed Approach	Baseline Approach
Balance Scale	7	**1.975**	3.555
	9	**2.980**	4.077
	11	**2.678**	5.003
Vehicle Silhouettes	5	**3.181**	3.889
	7	**3.262**	5.211
	9	**3.327**	6.400
	11	**2.848**	7.747
Car Evaluation	5	**5.756**	6.951
	7	**8.209**	9.213
	9	**9.367**	11.977
	11	**10.904**	14.278

**Table 6:** Decision templates for the Balance Scale dataset (7 local tables) for the proposed and baseline approaches. The values in the columns *p*(class) represent the averaged class membership probabilities $\mu_{j,i}\left(x\right)$ for each local model.

Proposed Approach				
**Decision Template**	**Local Model**	***p*** **(B)**	***p*** **(L)**	***p*** **(R)**
$DT_{B}$	$CT_{1}^{\mathrm{aggr}}$	0.588	0.235	0.176
	$CT_{2}^{\mathrm{aggr}}$	0.206	0.529	0.265
	$CT_{3}^{\mathrm{aggr}}$	0.294	0.235	0.471
	$CT_{4}^{\mathrm{aggr}}$	0.235	0.382	0.382
$DT_{L}$	$CT_{1}^{\mathrm{aggr}}$	0.030	0.950	0.020
	$CT_{2}^{\mathrm{aggr}}$	0.055	0.806	0.139
	$CT_{3}^{\mathrm{aggr}}$	0.134	0.776	0.090
	$CT_{4}^{\mathrm{aggr}}$	0.050	0.866	0.085
$DT_{R}$	$CT_{1}^{\mathrm{aggr}}$	0.020	0.025	0.955
	$CT_{2}^{\mathrm{aggr}}$	0.054	0.153	0.792
	$CT_{3}^{\mathrm{aggr}}$	0.134	0.079	0.787
	$CT_{4}^{\mathrm{aggr}}$	0.084	0.104	0.812
**Baseline Approach**				
**Decision Template**	**Local Model**	***p*** **(B)**	***p*** **(L)**	***p*** **(R)**
$DT_{B}$	$CT_{1}$	0.206	0.529	0.265
	$CT_{2}$	0.324	0.324	0.353
	$CT_{3}$	0.235	0.382	0.382
	$CT_{4}$	0.324	0.382	0.294
	$CT_{5}$	0.235	0.265	0.500
	$CT_{6}$	0.265	0.529	0.206
	$CT_{7}$	0.294	0.235	0.471
$DT_{L}$	$CT_{1}$	0.055	0.806	0.139
	$CT_{2}$	0.104	0.761	0.134
	$CT_{3}$	0.050	0.866	0.085
	$CT_{4}$	0.060	0.871	0.070
	$CT_{5}$	0.040	0.701	0.259
	$CT_{6}$	0.060	0.886	0.055
	$CT_{7}$	0.134	0.776	0.090
$DT_{R}$	$CT_{1}$	0.054	0.153	0.792
	$CT_{2}$	0.069	0.114	0.817
	$CT_{3}$	0.084	0.104	0.812
	$CT_{4}$	0.074	0.084	0.842
	$CT_{5}$	0.099	0.030	0.871
	$CT_{6}$	0.059	0.158	0.782
	$CT_{7}$	0.134	0.079	0.787

**Table 7:** Decision templates for the Vehicle Silhouettes dataset (5 local tables) for the proposed and baseline approaches. The values in the columns *p*(class) represent the averaged class membership probabilities $\mu_{j,i}\left(x\right)$ for each local model.

Proposed Approach					
**Decision Template**	**Local Model**	***p*** **(Bus)**	***p*** **(opel)**	***p*** **(saab)**	***p*** **(van)**
$DT_{bus}$	$CT_{1}^{\mathrm{aggr}}$	0.918	0.014	0.041	0.027
	$CT_{2}^{\mathrm{aggr}}$	0.856	0.027	0.110	0.007
	$CT_{3}^{\mathrm{aggr}}$	0.836	0.075	0.041	0.048
	$CT_{4}^{\mathrm{aggr}}$	0.877	0.021	0.062	0.041
$DT_{opel}$	$CT_{1}^{\mathrm{aggr}}$	0.043	0.713	0.189	0.055
	$CT_{2}^{\mathrm{aggr}}$	0.085	0.591	0.268	0.055
	$CT_{3}^{\mathrm{aggr}}$	0.018	0.530	0.372	0.079
	$CT_{4}^{\mathrm{aggr}}$	0.043	0.585	0.341	0.030
$DT_{saab}$	$CT_{1}^{\mathrm{aggr}}$	0.053	0.267	0.600	0.080
	$CT_{2}^{\mathrm{aggr}}$	0.053	0.287	0.580	0.080
	$CT_{3}^{\mathrm{aggr}}$	0.053	0.340	0.507	0.100
	$CT_{4}^{\mathrm{aggr}}$	0.080	0.307	0.567	0.047
$DT_{van}$	$CT_{1}^{\mathrm{aggr}}$	0.023	0.045	0.023	0.909
	$CT_{2}^{\mathrm{aggr}}$	0.068	0.076	0.045	0.811
	$CT_{3}^{\mathrm{aggr}}$	0.030	0.008	0.076	0.886
	$CT_{4}^{\mathrm{aggr}}$	0.008	0.121	0.038	0.833
**Baseline Approach**					
**Decision Template**	**Local Model**	***p*** **(Bus)**	***p*** **(opel)**	***p*** **(saab)**	***p*** **(van)**
$DT_{bus}$	$CT_{1}$	0.863	0.000	0.096	0.041
	$CT_{2}$	0.877	0.021	0.062	0.041
	$CT_{3}$	0.836	0.075	0.041	0.048
	$CT_{4}$	0.856	0.027	0.110	0.007
	$CT_{5}$	0.911	0.021	0.027	0.041
$DT_{opel}$	$CT_{1}$	0.024	0.689	0.232	0.055
	$CT_{2}$	0.043	0.585	0.341	0.030
	$CT_{3}$	0.018	0.530	0.372	0.079
	$CT_{4}$	0.085	0.591	0.268	0.055
	$CT_{5}$	0.079	0.549	0.329	0.043
$DT_{saab}$	$CT_{1}$	0.027	0.460	0.447	0.067
	$CT_{2}$	0.080	0.307	0.567	0.047
	$CT_{3}$	0.053	0.340	0.507	0.100
	$CT_{4}$	0.053	0.287	0.580	0.080
	$CT_{5}$	0.087	0.247	0.593	0.073
$DT_{van}$	$CT_{1}$	0.038	0.038	0.083	0.841
	$CT_{2}$	0.008	0.121	0.038	0.833
	$CT_{3}$	0.030	0.008	0.076	0.886
	$CT_{4}$	0.068	0.076	0.045	0.811
	$CT_{5}$	0.068	0.114	0.061	0.758

**Table 8:** Decision templates for the Car Evaluation dataset (7 local tables) for the proposed and baseline approaches. The values in the columns *p*(class) represent the averaged class membership probabilities $\mu_{j,i}\left(x\right)$ for each local model.

Proposed Approach					
**Decision Template**	**Local Model**	***p*** **(acc)**	***p*** **(Good)**	***p*** **(unacc)**	***p*** **(vgood)**
$DT_{acc}$	$CT_{1}^{\mathrm{aggr}}$	0.704	0.019	0.277	0.000
	$CT_{2}^{\mathrm{aggr}}$	0.692	0.017	0.280	0.011
	$CT_{3}^{\mathrm{aggr}}$	0.652	0.017	0.314	0.017
	$CT_{4}^{\mathrm{aggr}}$	0.599	0.041	0.346	0.015
	$CT_{5}^{\mathrm{aggr}}$	0.599	0.043	0.325	0.033
	$CT_{6}^{\mathrm{aggr}}$	0.665	0.032	0.281	0.022
$DT_{good}$	$CT_{1}^{\mathrm{aggr}}$	0.229	0.510	0.219	0.042
	$CT_{2}^{\mathrm{aggr}}$	0.219	0.552	0.208	0.021
	$CT_{3}^{\mathrm{aggr}}$	0.104	0.549	0.285	0.062
	$CT_{4}^{\mathrm{aggr}}$	0.292	0.458	0.229	0.021
	$CT_{5}^{\mathrm{aggr}}$	0.188	0.469	0.240	0.104
	$CT_{6}^{\mathrm{aggr}}$	0.104	0.490	0.240	0.167
$DT_{unacc}$	$CT_{1}^{\mathrm{aggr}}$	0.093	0.010	0.890	0.007
	$CT_{2}^{\mathrm{aggr}}$	0.097	0.009	0.882	0.011
	$CT_{3}^{\mathrm{aggr}}$	0.077	0.009	0.896	0.018
	$CT_{4}^{\mathrm{aggr}}$	0.119	0.019	0.845	0.017
	$CT_{5}^{\mathrm{aggr}}$	0.103	0.014	0.870	0.014
	$CT_{6}^{\mathrm{aggr}}$	0.135	0.005	0.836	0.024
$DT_{vgood}$	$CT_{1}^{\mathrm{aggr}}$	0.122	0.000	0.278	0.600
	$CT_{2}^{\mathrm{aggr}}$	0.022	0.044	0.278	0.656
	$CT_{3}^{\mathrm{aggr}}$	0.022	0.067	0.144	0.767
	$CT_{4}^{\mathrm{aggr}}$	0.311	0.044	0.267	0.378
	$CT_{5}^{\mathrm{aggr}}$	0.000	0.222	0.156	0.622
	$CT_{6}^{\mathrm{aggr}}$	0.111	0.233	0.167	0.489
**Baseline Approach**					
**Decision Template**	**Local Model**	***p*** **(acc)**	***p*** **(Good)**	***p*** **(unacc)**	***p*** **(vgood)**
$DT_{acc}$	$CT_{1}$	0.599	0.041	0.346	0.015
	$CT_{2}$	0.558	0.024	0.392	0.026
	$CT_{3}$	0.665	0.032	0.281	0.022
	$CT_{4}$	0.550	0.056	0.394	0.000
	$CT_{5}$	0.654	0.071	0.268	0.007
	$CT_{6}$	0.617	0.098	0.285	0.000
	$CT_{7}$	0.599	0.043	0.325	0.033
$DT_{good}$	$CT_{1}$	0.292	0.458	0.229	0.021
	$CT_{2}$	0.271	0.458	0.271	0.000
	$CT_{3}$	0.104	0.490	0.240	0.167
	$CT_{4}$	0.208	0.479	0.271	0.042
	$CT_{5}$	0.271	0.521	0.125	0.083
	$CT_{6}$	0.229	0.444	0.243	0.083
	$CT_{7}$	0.188	0.469	0.240	0.104
$DT_{unacc}$	$CT_{1}$	0.119	0.019	0.845	0.017
	$CT_{2}$	0.083	0.006	0.901	0.009
	$CT_{3}$	0.135	0.005	0.836	0.024
	$CT_{4}$	0.164	0.015	0.812	0.008
	$CT_{5}$	0.112	0.030	0.849	0.009
	$CT_{6}$	0.133	0.015	0.837	0.014
	$CT_{7}$	0.103	0.014	0.870	0.014
$DT_{vgood}$	$CT_{1}$	0.311	0.044	0.267	0.378
	$CT_{2}$	0.178	0.222	0.289	0.311
	$CT_{3}$	0.111	0.233	0.167	0.489
	$CT_{4}$	0.178	0.000	0.289	0.533
	$CT_{5}$	0.311	0.044	0.133	0.511
	$CT_{6}$	0.200	0.111	0.156	0.533
	$CT_{7}$	0.000	0.222	0.156	0.622

**Table 9:** Results of classification accuracy (Acc), balanced accuracy (BAcc), precision (Prec.), recall, F-measure (F-m.), and geometric mean (G-mean) for the coalition approach using decision rule models across datasets and dispersion levels, as reported in [30].

Dataset	# Local Tables	Best Rule Induction Method	Acc/BAcc/Prec./Recall/F-m./G-Mean	ΔAcc
Balance Scale	7	Exh/Gen	0.745/0.742/0.890/0.745/0.795/0.841	−0.006
	9	Exh/Gen	0.686/0.681/0.856/0.686/0.745/0.798	0.000
	11	Exh/Gen	0.697/0.670/0.863/0.697/0.756/0.806	**+0.042**
Vehicle Silhouettes	5	Exh	0.713/0.700/0.711/0.713/0.706/0.803	**+0.039**
	7	Exh	0.701/0.686/0.697/0.701/0.693/0.796	**+0.008**
	9	Exh	0.701/0.695/0.707/0.701/0.693/0.797	**+0.079**
	11	Gen	0.701/0.690/0.706/0.701/0.696/0.796	−0.012
Car Evaluation	5	Gen	0.744/0.674/0.761/0.744/0.750/0.745	**+0.023**
	7	Exh/Gen	0.748/0.641/0.760/0.748/0.752/0.743	**+0.017**
	9	Exh/Gen	0.765/0.726/0.786/0.765/0.772/0.774	−0.014
	11	Exh/Gen	0.765/0.758/0.790/0.765/0.773/0.783	−0.008

## Data Availability

Publicly available datasets were analyzed in this study. These data can be found at the UCI Machine Learning Repository [37].
